# Decrease in Hospitalizations for Pneumonia in Children under Five Years of Age in an Indian Reservation in Panama after the Introduction of the Heptavalent Pneumococcal Conjugate Vaccine (PCV7)

**DOI:** 10.1155/2013/514578

**Published:** 2013-05-09

**Authors:** Javier Nieto Guevara, Carlos Daza, Rebecca Smith

**Affiliations:** ^1^Instituto Conmemorativo Gorgas de Estudios de la Salud, Pediatric Infectious Diseases, Panama; ^2^Hospital Materno Infantil José Domingo de Obaldía, Panama

## Abstract

This study quantifies the impact of Heptavalent-Pneumococcal Conjugate Vaccine (PCV7) in Panama on indigenous children younger than 5 years old, based on clinical pneumonia cases. This study demonstrates a significant 41.2% reduction in hospitalizations and 38.6% reduction in referrals for pneumonia following the introduction of PCV7. Burden of disease from pneumonia appears reduced in the ≤12-month- and 13-to-24-month-old groups.

## 1. Introduction

*Streptococcus pneumonae* is a Gram-positive, anaerobic, catalase-negative bacterium. It produces a polysaccharide capsule, that is used for serotyping; more than 90 immunologically distinct serotypes have been identified. They form 46 serogroups from which fewer than 20 serotypes account for most of cases of the disease caused by *S. pneumoniae*.

Pneumococcal diseases have great impacts, and many of the 1.9 million deaths reported worldwide from respiratory illnesses in children younger than 5 years of age are due to *S. pneumoniae* [1]. In Latin America, it is estimated that 60,000 deaths a year are caused by respiratory infections, representing 3.2% of all deaths worldwide [1]. Approximately 14% of the children in that region die from acute respiratory illnesses, compared with 11% in Europe and 22% in Africa [1]. Between 12,000 and 28,000 deaths in Latin America are due to pneumococcal infections [2].

Because of the high social and economical burden of pneumococcal diseases, the World Health Organization (WHO) has recognized these infections as one of the main causes of morbidity and mortality worldwide and accepted that prevention has a key role on diminishing such burdens. Vaccination is the main preventive strategy [3], and in 2006, the WHO recommended vaccination against *S. pneumonie* as a priority [4].

On December 1, 2008, the Panamanian Government introduced the heptavalent pneumococcal conjugated vaccine (Prevenar, PCV7, Wyeth Pharmaceuticals Inc., a subsidiary of Pfizer Pharmaceuticals Inc, Philadelphia, PA USA) for all children ≤9 years of age in the indigenous Ngöbe-Buglé Reservation. PCV 7 vaccine was administered in a four-dose schedule (2, 4, 6, and 12–15 months of age) to children less than two years old, with a single catch-up dose administered to all children between 2 and 9 years of age.

The Ngöbe-Buglé Region is located in the west of the Republic of Panama and comprises 6,994.06 km^2^, with a population of 122,423 inhabitants, including 25,178 children aged less than 5 years [5]. The poverty level in the region is 93.4%, compared to the 37.3% national average; malnutrition ranges from 35% to 63% (24% national average); and infant mortality is 22.6 per 1,000 live births (national rate of 15.4/1,000) [6].

The goal of this study was to assess the impact of the PCV7 vaccination under regular conditions on the incidence of clinical pneumonia in a high risk and impoverished population. We analyzed the effect of PCV7 introduction on hospitalizations and healthcare center transfers to the regional hospital due to pneumonia prior to and following PCV7 introduction in the Ngöbe-Buglé Region. 

## 2. Materials and Methods

This observational study was carried out at Hospital Materno Infantil José Domingo de Obaldía (HMIJDDO), a second-level reference hospital with 278 pediatric beds that serves the pediatric patients in the Ngöbe-Buglé Reservation, which correspond to 70% of the hospital's pediatric patients (Information provided by Medical Registry Department of Children Hospital at Jose Domingo de Obaldia).

We analyzed two periods: P1, prior to the PCV7 introduction (June 2007 to November 2008), and P2, after the introduction of the vaccine (December 2008 to May 2010), searching for hospitalizations and referrals for pneumonia in children <5 years of age in both periods. Subjects were stratified as ≤12 months, 13–24 months, 25–36 months, and 37–60 months of age.

Pneumonia hospitalization data were obtained from medical records in the statistics department at HMIJDDO based on a discharge diagnosis of pneumonia given by the treating physician and coded according to ICD 9-CM. 

From the medical record, we also obtained relevant information such as age, sex, place of origin (if the patient came from an indigenous reservation), referral site (if the patient was referred to HMIJDDO for management from another health institution in the area), date of entry and date of discharge, and status at discharge (improved or deceased). The inclusion criteria were boys and girls, 2 to 60 months of age, from the indigenous area, with a discharge diagnosis of pneumonia. The exclusion criterion was the lack of needed information on the medical record. The immunization status was obtained from the Region's Immunization Program. 

The statistical analysis was performed through Epi-Info (V3.2-CDC, Atlanta) using Yates' chi-squared test for categorical variables as well as Student's *t* analyses for continuous variables. Results were expressed in terms of pneumonia hospitalizations and referrals/10,000 admissions and percentage reductions in terms of absolute number of cases. A confidence interval of 95% and *P* ≤ 0.05 were considered statistically significant. 

## 3. Results

A total of 179 patients with pneumonia were hospitalized during P1 versus 99 patients in P2. There was no statistically significant difference in relation to age, sex, length of stay, and prognosis of the subjects involved. The vaccine coverage rates in 2009 in children <24 months were 91%, 43%, 17%, and 57% for one, two, three, and booster doses, respectively. The coverage rate for the catch-up dose for children between 5 and 9 years was 11%. In 2010, vaccine coverage rates were 98%, 55%, 30%, and 7% for one, two, three, and booster doses, respectively. The coverage rate for the catch-up dose in children between 5 and 9 years was 4%. 

**Figure 1:**
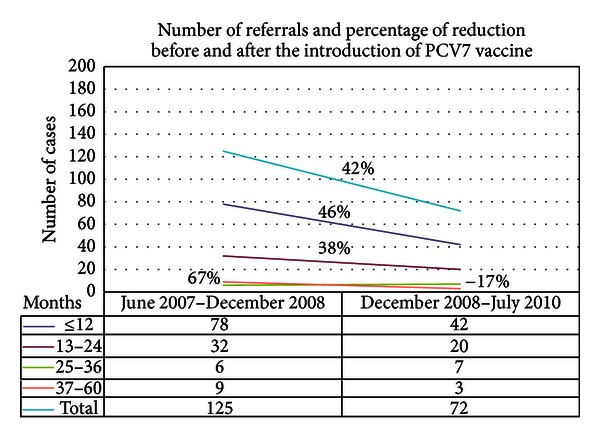
Number of referrals and percentage of reduction before (Jun 2007–Dec 2008) and after (Dec 2008–Jul 2010) PCV7 introduction, according to patient age in months.

The universal incidence of hospitalizations per 10,000 admissions decreased from 548.0 (95% CI: 504, 594) in P1 to 322.4 (95% CI: 289, 358) in P2, a significant 41.2% reduction. There was also a statistically significant decrease of 38,6% in referrals for pneumonia following introduction of PCV7 (637.7 (95% CI: 590, 686) to 391.3 (95% CI: 354, 430)). This decrease was the highest in ≤12-month- and 13–24-month groups (46% and 38%, resp.) (Figure 1). 

## 4. Discussion

This study demonstrates a significant 41.2% reduction in hospitalizations and 38.6% reduction in referrals for pneumonia in indigenous children younger than 5 years of age following the introduction of PCV7. Our data are compatible with those reported by other studies from Uruguay, the USA and Poland [3, 7, 8]. In Uruguay, a 56% decrease in episodes of community-acquired pneumonia followed introduction of PCV7 in children less than 2 years of age with a 2+1 schedule [7]. In the USA, a reduction (39%) in pneumonia cases after PCV7 vaccines was observed for children under 2 years under a 3+1 schedule [3]. In Poland, the reduction in children under 2 years of age was 65% (pneumonia radiologically confirmed) [8]. Although studies have used different methodological parameters, all concur that immunization with PCV7 helps decrease clinical pneumonia due to *S. pneumoniae*. 

In our study, the burden of disease from pneumonia appears primarily in the ≤12-month-old and 13 to 24 month-old groups (Figure 1), which are those with the greatest morbidity and mortality [5]. Our study was limited by its observational nature and because we could not assess changes in chest radiographs, due to the lack of systematic recording of radiographic results. Our small sample size for children ≥2 years of age can be associated with a tendency of no decrease or increase of pneumonia episodes, a finding that needs to be cautiously interpreted. A greater sample size could yield a more objective conclusion with respect to this finding.

Another explanation for the greater impact of the immunization program in children younger than 1 year of age is that this group had better vaccine coverage than other age groups. Coverage in children under one year of age was 43% for second doses in 2009 and 55% in 2010. This data further adds to the uncertainty of how many doses are needed to adequately protect and achieve a herd effect [5, 9]. This information is important to help analyze the real impact of immunization in marginal populations under usual conditions and reinforces the fact that vaccination is important in reducing racial and social inequities.

Since this is a retrospective, observational study, we cannot be certain that the tendency towards a decrease in hospitalization episodes for pneumonia is solely due to the vaccine's effect. There was no change in criteria for hospitalization or for referrals from other healthcare centers in the region, or in access to healthcare services; thus, we believe that the decrease is real and potentially related to PCV7 introduction. A prospective epidemiological follow-up should be performed to confirm these results. 

To our knowledge, this is the first Latin American study in an indigenous population under usual vaccination conditions with PCV7, in which reductions in hospitalizations and referrals for pneumonia in children younger than 5 years of age were demonstrated, with greatest effects in children younger than 1 year of age. Analysis of serotype prevalence after vaccine implementation will be necessary, but these findings support the concept expressed at the 63rd World Health Assembly that infant mortality can be reduced by two-thirds based on prompt treatment and prevention of pneumonia. Panama introduced PCV13 in August 2011. Active surveillance will be important in evaluating the impact of this vaccine on the pediatric population.
